# Risk factors for bloodstream infection among patients admitted to an intensive care unit of a tertiary hospital of Shanghai, China

**DOI:** 10.1038/s41598-024-63594-8

**Published:** 2024-06-04

**Authors:** Yingchao Cui, Changlin Yi, Chaomin Zhang, Chihui Yang, Xinyi Wang, Wenkai Chen, Yibing Peng, Jing Dai

**Affiliations:** Department of Laboratory Medicine, Ruijin Hospital, Shanghai Jiao Tong University School of Medicine, No. 197 Second Ruijin ER Road, Shanghai, 200025 China

**Keywords:** Bloodstream infection, Risk factors, Tigecycline, Platelet count, Intensive care unit, Risk factors, Epidemiology, Outcomes research

## Abstract

Blood flow infections (BSIs) is common occurrences in intensive care units (ICUs) and are associated with poor prognosis. The study aims to identify risk factors and assess mortality among BSI patients admitted to the ICU at Shanghai Ruijin hospital north from January 2022 to June 2023. Additionally, it seeks to present the latest microbiological isolates and their antimicrobial susceptibility. Independent risk factors for BSI and mortality were determined using the multivariable logistic regression model. The study found that the latest incidence rate of BSI was 10.11%, the mortality rate was 35.21% and the mean age of patients with BSI was 74 years old. Klebsiella pneumoniae was the predominant bacterial isolate. Logistic multiple regression revealed that tracheotomy, tigecycline, gastrointestinal bleeding, shock, length of hospital stay, age and laboratory indicators (such as procalcitonine and hemoglobin) were independent risk factors for BSI. Given the elevated risk associated with use of tracheotomy and tigecycline, it underscores the importance of the importance of cautious application of tracheostomy and empirical antibiotic management strategies. Meanwhile, the independent risk factors of mortality included cardiovascular disease, length of hospital stay, mean platelet volume (MPV), uric acid levels and ventilator. BSI patients exhibited a significant decrease in platelet count, and MPV emerged as an independent factor of mortality among them. Therefore, continuous monitoring of platelet-related parameters may aid in promptly identifying high-risk patients and assessing prognosis. Moreover, monitoring changes in uric acid levels may serve as an additional tool for prognostic evaluation in BSI patients.

## Introduction

The incidence of bloodstream infections (BSIs) caused by pathogens has been on the rise, emerging as one of the leading causes of morbidity in recent years, especially in intensive care units (ICUs)^1^. Studies conducted in North America, Europe and other developed countries indicate that the incidence ranged from 113 to 204 per 100,000 population^2,3^. Patients admitted to the ICUs typically present with complex and severe conditions, low immunity, and a heightened susceptibility to BSIs compared to other wards^4^. BSIs also increase ICU hospitalization time and healthcare costs. Consequently, significant research has focused on attempting to enhance patient survival rates and outcome through the prevention or early prediction of predisposing indicators.

In ICUs, the overall health status of patients, underlying diseases and comorbidities (such as multiple organ dysfunction), along with medical interventions adopted by medical staff (such as tracheostomy, blood transfusion, enteral feeding, etc.) may contribute to the increased incidence of BSI in ICU^5^. In addition, prolonged treatment with multiple antibiotics may lead to drug resistance of the pathogenic microorganisms colonized in the patient's body^6–8^. Current empirical treatments^9^ are not applicable across all regions for suspected BSI, thereby heightening the risk of adverse outcomes. Hence, it is crucial to update knowledge regarding the microbiological infection status of BSI pathogens, patient demographics, and medical interventions to support therapeutic guidelines. Additionally, the collective indication of laboratory indicators is also very important.

BSI is one of the most severe infections, often culminating in severe sepsis or septic shock among patients necessitating intensive care. In China, studies have revealed that the BSI burden rate in ICUs stands at 10.85%^10^. It is a huge challenge for clinical doctors, given its potential to trigger systemic infections associated with high mortality rates. Considering the living cost and the saturation of the city center, the population in the suburbs of Shanghai has grown rapidly in recent years. However, the descriptions of pathogen appearance and microbial characteristics in BSI patients of suburban ICUs are scarce. In the this study, we performed a retrospective study spanning the latest period in tertiary hospitals situated in the expansive suburban areas. Our objectives were to estimate the incidence of BSI, explore the efficacy of early intervention measures related to BSI occurrence, and explore early predictive value of laboratory indicators for early detection. Furthermore, we aimed to assess the mortality rate of patients with BSI and delineate its independent impact, thereby facilitating the selection of appropriate therapies and laboratory indicators for the prompt for early diagnosis and treatment of ICU patients with BSI.

## Methods

### Study design, case definitions, and data collection

We conducted a retrospective cross-sectional study on adult patients admitted to our ICU ward, an 30-bed department in Shanghai Ruijin Hospital north, from January 2022 to June 2023. Our hospital is a tertiary hospital located in the suburbs of Shanghai, with a permanent population of 1.8934 million. It is a first-line medical center in this area. All methods were performed in accordance with the relevant guidelines and regulations. This study was approved by the Ethics Committee of Ruijin Hospital, Shanghai Jiao Tong University School of Medicine. All related individuals gave their informed consent to paticipate. All patients aged 18 and above were admitted to the ICU during the study period and received clinical evaluation for blood culture testing by attending physicians. All patients meeting above criteria were assessed for inclusion, including 271 patients. In this study, a total of 71 BSI-positive patients and 200 BSI-negative patients were included. BSI was defined according to the CDC definitions^11^. BSIs were defined as the isolation of pathogenic organisms from blood culture test. Sepsis shock was evaluated by clinician according to the Sepsis-3^12^. Assessment II (APACHE II) score is used to calculate the severity of the disease within 24 h followingthe onset of BSI. We collected the basic information of cases, including demographic data (gender, age and mortality), disease factors (COVID-19, gastrointestinal, respiratory, cardiovascular, renal, neurologic, metabolic disorders and immunocompromise), treatment interventions before infections events (surgical intervention, enteral feeding, trauma, gastrointestinal bleeding, transfusion, dialysis therapy, central venous catheters, tracheotomy and ventilator) and other laboratory parameters when culturing blood samples.

### Culture and laboratory methods

Blood samples were cultured by Bact Alert 3D (bioMérieux, France) and identified by MALDI-TOF MS spectrometry (bioMérieux, France). For antimicrobial susceptibility testing, isolated strains were performed with the VITEK-2 compact system (bioMérieux, France) and Kirby–Bauer (K–B) paper diffusion method. Antibiotic susceptibility were defined based on the Clinical and Laboratory Standards Institute (CLSI)-M100 2021 guidelines.

### Statistical analyses

Data were analyzed by SPSS 23.0. The continuous variables were represented as median and interquartile ranges (IQRs) and the categorical variables were expressed as frequency counts. For categorical variables between groups, chi square test was used, and for continuous variables, Student’s t-test or the Kruskal–Wallis test were used. In analysis of risk factors for BSI occurrence and death, a multivariate logistic regression model was performed. Kaplan–Meier analyses was used to determine the relationship between certain treatments of patients and the occurrence of BSI as hospitalization time increases. A *p* value of < 0.05 was considered statistically significant.

## Results

### Patient population

**Figure 1:**
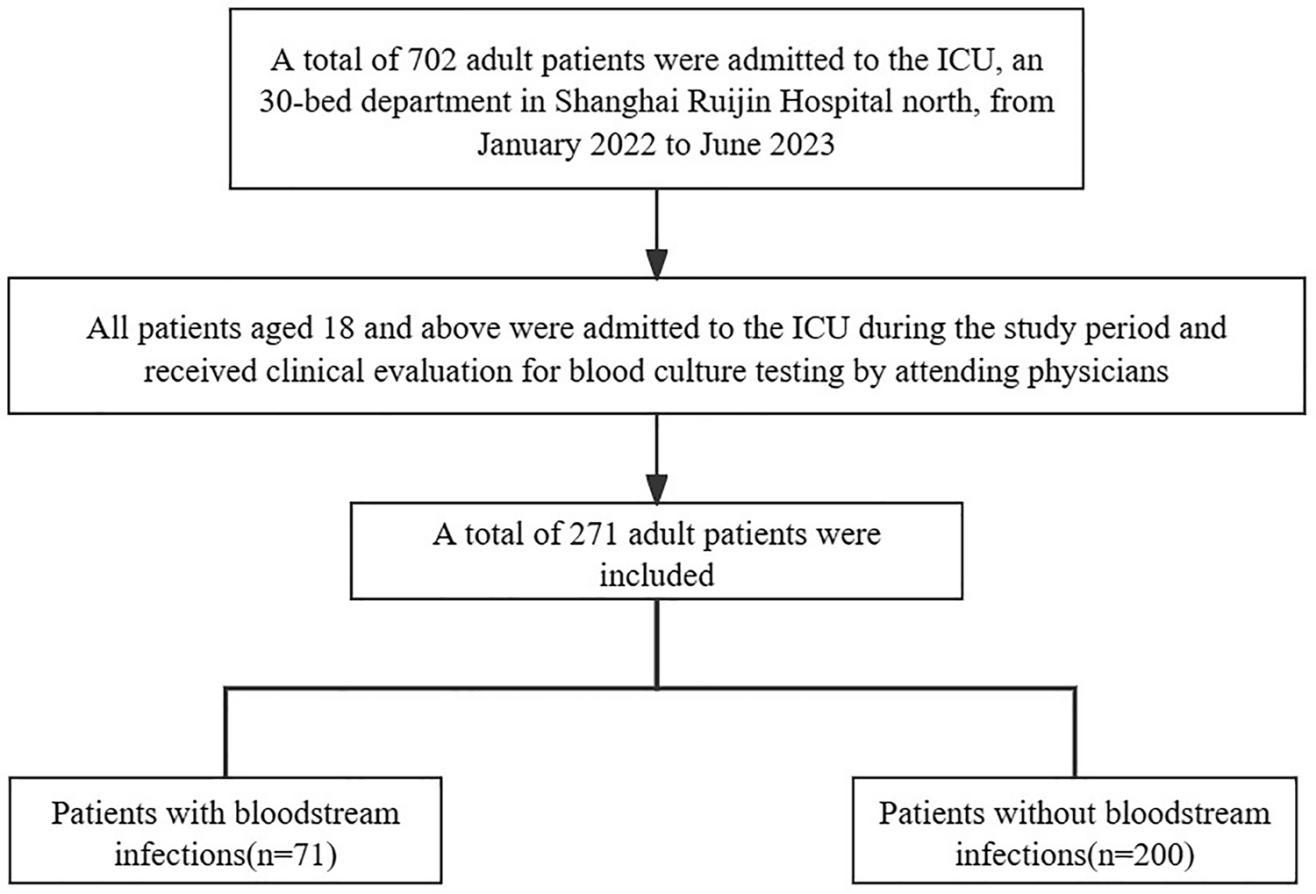
Flowchart of clinical study.

About 702 adult patients were admitted to the ICU during the study period. Of these, 271 patients were sent for blood culture test. 71 patients developed a bloodstream infection during hospitalization. The overall positivity of all blood cultures was 26.20% (71/271) and the overall prevalence of bloodstream infection was 10.11% (71/702) (Fig. 1). The mean age of patients with bloodstream infections was 74(65–84) years old, and 54 patients (76.06%) were men. The mean ages were 69.5(56–78) years in the patients without bloodstream infections in ICU ward and 136 patients (68.00%) were men. The median hospital stay for BSI patients was 30 days and significantly longer than that for non-BSI patients (*p* < 0.001) (Table 1).

**Table1:** Characteristics of patients admitted to ICU with univariate comparisons.

Patients characteristics	Bloodstream infection patients (n = 71) n(%)/median (IQR)	Without bloodstream infection patients (n = 200) n (%)/median (IQR)	Univariate analysis	
			OR (95% CI)	*p* value
Demographics				
Male	54 (76.06)	136 (68.00)	1.004 (0.555–1.817)	0.990
Age	74 (65–84)	69.5 (56–78)	–	0.072
Fever (≥ 38 °C)	40 (56)	123 (62)	0.808 (0.467–1.398)	0.445
Chronic comorbidity				
COVID-19	12 (17)	21 (11)	1.734 (0.804–3.736)	0.156
Gastrointestinal	43 (61)	95 (48)	1.697 (0.978–2.945)	0.059
Respiratory	44 (62)	105 (53)	1.474 (0.848–2.565)	0.168
Cardiovascular	31 (44)	81 (41)	1.139 (0.659–1.968)	0.642
Renal	28 (39)	83 (42)	0.918 (0.528–1.596)	0.761
Neurologic	23 (32)	101 (51)	0.470 (0.266–0.830)	0.009*
Metabolic disorders	45 (63)	118 (59)	1.203 (0.688–2.104)	0.517
Immunocompromise	12 (16.9)	43 (21.5)	0.743 (0.366–1.505)	0.408
Severity at BSI onset				
Shock	46 (65)	83 (42)	2.594 (1.478–4.552)	0.001*
APACHE II score > 15	47 (66.2)	130 (65)	1.054 (0.596–1.867)	0.856
Treatment interventions before infections events				
Surgical intervention	33 (46)	75 (38)	1.436 (0.830–2.482)	0.194
Enteral feeding	44 (62)	151 (76)	0.529 (0.297–9.942)	0.029*
Trauma	6 (8)	20 (10)	0.831 (0.320–2.160)	0.703
Gastrointestinal bleeding	15 (21)	19 (10)	2.552 (1.217–5.350)	0.011*
Transfusion	39 (55)	88 (44)	1.551 (0.900–2.674)	0.113
Dialysis therapy	29 (41)	54 (27)	1.867 (1.059–3.291)	0.03*
Tracheotomy	25 (35.2)	31 (15.5)	2.963 (1.595–5.505)	< 0.001*
Ventilator	47 (66.2)	145 (72.5)	0.743 (0.415–1.329)	0.315
Central venous catheter	53 (74.6)	132 (66)	1.517 (0.825–2.790)	0.179
Outcome				
Length of stay				
Total hospital stay (d)	30 (13–53)	21 (11–35)	–	< 0.001*
ICU stay (d)	21 (8–41)	15 (8–26)	–	< 0.001*
Mortality	25 (35.21)	40 (20)	2.174 (1.196–3.952)	0.01*
Laboratory parameter				
Alkaline phosphatase^a^	102.5 (75.75–173.5)	81 (58–113)	–	0.005*
Alanine transaminase^a^	34 (15–85)	27 (13–55.75)	–	0.951
Aspartate aminotransferase^a^	44 (26–89)	39 (20–80)	–	0.64
γ-glutamyl transpeptadase^a^	61 (40–121)	33 (20–87.5)	–	0.013*
Total bilirubin level^b^	22.9 (15.2–56)	17.3 (10.93–30.45)	–	0.095
Direct bilirubin^b^	8.2 (4.3–31.9)	5.3 (2.93–11.48)	–	0.099
Total biliary acid^b^	5.55 (3.18–16.58)	4.1 (2.3–8.5)	–	0.263
Cholyglycine^b^	3.1 (1.8–9.9)	2 (1.5–3.9)	–	0.067
Total protein^c^	59.5 (51.5–66.25)	58 (52.25–63)	–	0.298
Serum creatinine level^b^	105 (71–154)	92 (66–158.75)	–	0.975
Brain natriuretic peptide^d^	698 (237.75–1599.25)	273 (104.5–856.5)	–	0.02*
Procalcitonin^e^	6.34 (1.23–52)	0.69 (0.24–3.85)	–	< 0.001*
Hematocrit	0.27 (0.21–0.34)	0.3 (0.25–0.34)	–	0.022*
Hemoglobin^c^	87 (70–113)	98 (82–115.75)	–	0.008*
Leukocyte count^f^	9.3 (6.54–18.12)	11 (7.41–14.29)	–	0.788
Neutrophil count^f^	8.16 (5.62–17.21)	9.41 (6.18–12.41)	–	0.328
Lymphocyte count^f^	0.58 (0.33–0.98)	0.69 (0.45–1.04)	–	0.547
Monocyte count^f^	0.3 (0.17–0.62)	0.48 (0.28–0.76)	–	0.08
Mean platelet volume (fl)	10.8 (9.88–12.13)	10.3 (9.4–11.3)	–	0.003*
Platelet count^f^	113 (44–203)	151 (102–220)	–	0.023*
Erythrocyte count^f^	2.91 (2.23–3.66)	3.24 (2.68–3.89)	–	0.017*
CRP (mg/L)	143.5 (93–200)	96.5 (40.25–164.75)	–	< 0.001*
Antibiotic usage				
Antifungal agents	30 (42)	123 (62)	0.458 (0.264–0.794)	0.005*
Fluoroquinolone	24 (34)	71 (36)	0.928 (0.524–1.642)	0.797
Peptide antibiotic	30 (42)	110 (55)	0.599 (0.346–1.035)	0.065
Cephalosporin	35 (49)	153 (77)	0.299 (0.169–0.527)	< 0.001*
Carbapenem	52 (73)	152 (76)	0.864 (0.466–1.603)	0.643
Tigecycline	13 (18)	14 (7)	2.978 (1.324–6.697)	0.006*
Linezolid	21 (30)	75 (38)	0.700 (0.390–1.256)	0.231

^a^ U/L ^b^μmol/L ^c^g/L ^d^pg/ml ^e^ng/mL ^f^10^9^/L.

*p < 0.05.

### Clinical characteristics of patients with bloodstream infection included in this study

As shown in Table 1, univariate analysis was used to explore the differences in clinical features, diagnosis, and treatment between patients with or without bloodstream infection. bloodstream infection patients are more prone to shock (OR, 2.594; 95% CI 1.478–4.552; *p* = 0.001) compared to patients without bloodstream infection. Compared with non bloodstream infection patients, patients with BSI more frequently occurred gastrointestinal bleeding (21% vs. 10%; OR, 2.552; 95% CI 1.217–5.350; *p* = 0.011) and adoptted dialysis therapy (41% vs. 27%; OR, 1.867; 95% CI 1.059–3.291; *p* = 0.03). 25 out of 71 patients with BSI underwent tracheostomy, showing a significant difference between the two groups (OR 2.963; 95% CI 1.595–5.505; *p* < 0.001). Furthermore, a larger proportion of patients were administered enteral feeding (OR 0.529; 95% CI 0.297–9.942; *p* = 0.029), and has shown significant protective effects in BSI group.

For antibiotic treatment, antifungal agent usage (*p* = 0.005) and cephalosporin (*p* < 0.001) were more frequently found in non-BSI patients, while tigecycline was a risk factor (OR 0.013; 95% CI 0–0.512; *p* = 0.021) for BSI patients. For laboratory tests,we found that BSI patients have significantly higher ALP, GGT, BNP, PCT, MPV, CRP levels and lower Hct, hemoglobin levels, platelet count and erythrocyte count than patientswithout BSI.

**Figure 2:**
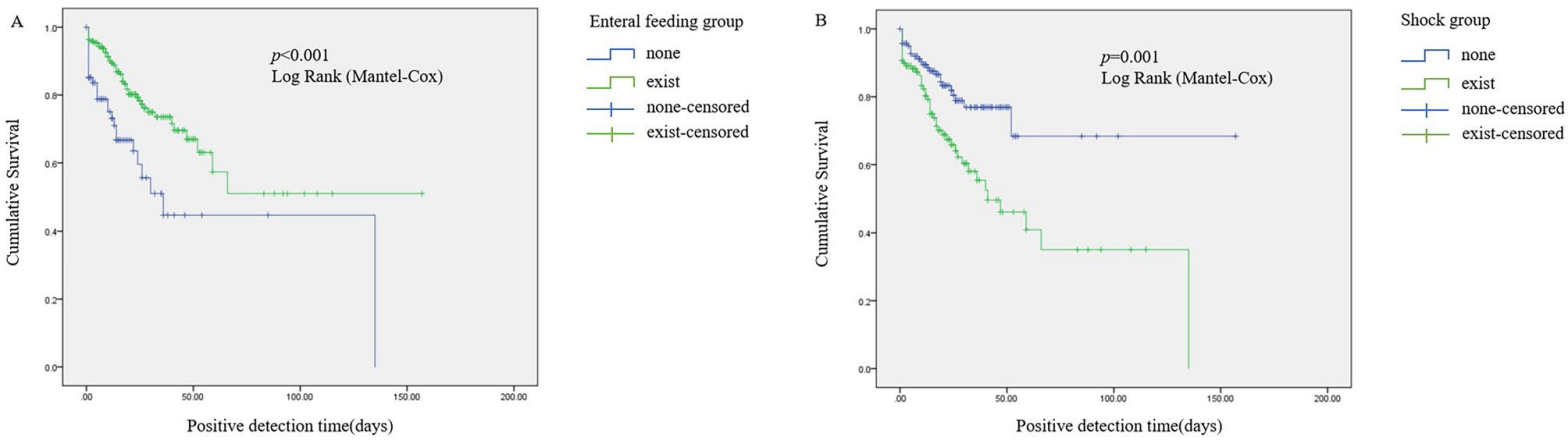
Probability of occurrence for the patient with bloodstream infection by therapy. (**A**) Enteral feeding group; (**B**) Shock group.* p* values were calculated using log Rank-Mantel. *p* < 0.05 means statistically significant.

Survival analysis was conducted to explore the impact of these univariate variables on the occurrence of BSI. Notably, the BSI rate of patients who used enteral feeding (*p* < 0.001) during hospitalization was significantly lower than that of patients without enteral feeding over time. And the occurrence of shock in patients had markedly increased the BSI rate over time (Fig. 2).

As illustrated in Table 2, the factors significantly correlated with BSI were adjusted for multivariate model analysis. We found that the use of tracheotomy (OR 7.603; 95% CI 2.944–19.631; *p* < 0.001), shock (OR 3.526; 95% CI 1.432–8.686; *p* = 0.006), gastrointestinal bleeding (OR 3.274; 95% CI 1.171–9.149; *p* = 0.024) and tigecycline (OR 2.952; 95% CI 1.009–8.636; *p* = 0.048) can particularly promote the occurrence of BSI. Meanwhile, the use of cephalosporin (OR 0.154; 95% CI 0.063–0.376; *p* < 0.001), antifungal agents (OR 0.149; 95% CI 0.058–0.281; *p* < 0.001) and peptide antibiotic (OR 0.220; 95% CI 0.088–0.533; *p* = 0.001) were protective factors for BSI. In addition, hospital stay, age, PCT levels and hemoglobin levels and the above seven indicators were independent indicators of developing BSI in ICU acquired patients.

**Table 2:** Characteristics of patients admitted to ICU with multivariable logistic regression analysis.

Patients Characteristics	Multivariable logistic regression Analysis	
	OR (95% CI)	*p* value
Tracheotomy	7.603 (2.944–19.631)	< 0.001
Shock	3.526 (1.432–8.686)	0.006
Gastrointestinal bleeding	3.274 (1.171–9.149)	0.024
Tigecycline	2.952 (1.009–8.636)	0.048
Age	1.038 (1.010–1.067)	0.008
Hospital stay	1.031 (1.014–1.049)	< 0.001
Procalcitonin^b^	1.021 (1.088–1.034)	0.001
Antifungal agents	0.149 (0.058–0.281)	< 0.001
Cephalosporin	0.154 (0.063–0.376)	< 0.001
Peptide antibiotic	0.220 (0.088–0.533)	0.001
Hemoglobin^a^	0.977 (0.959–0.995)	0.012

^a^g/L ^b^ng/mL.

### Characteristics of infectious pathogens

**Figure 3:**
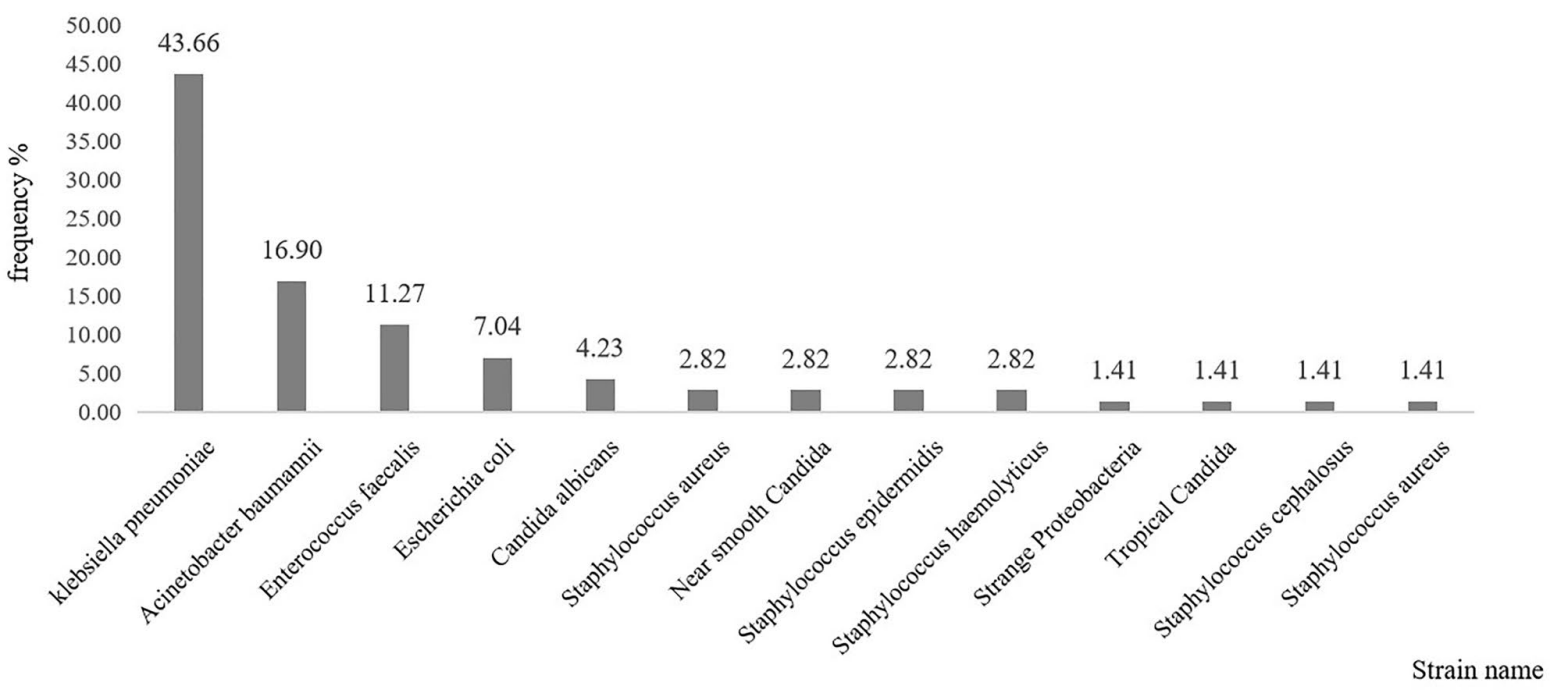
Distribution of pathogens observed in patients with bloodstream infection admitted to the intensive care unit.

The pathogens of BSI are presented in Fig. 3. The median time from admission to the first BSI was 12 days. The common causative pathogens were Gram-negative bacteria (49, 69%), Gram-positive bacteria (16, 22.5%), and fungi (n = 6, 8.5%) in the patients with BSI. The highest proportion of Gram-negative bacteria was Klebsiella pneumoniae (31/49, 63.3%), and then was Acinetobacter baumannii (12/49, 24.5%), and then was Escherichia coli (5/49, 10.2%). Gram-positive pathogens were mainly Enterococcus faecalis (8/16, 50%).

### Characteristics of clinical isolates of Klebsiella pneumoniae

In vitro susceptibility test, all Klebsiella Pneumoniae was sensitive to polymyxin-b, 67.7% were susceptible to amikacin and tigecycline, and 54.8% were susceptible to gentamicin. The positive result of extended spectrum Beta-Lactamases detection was 96.8%. Additionally, we also conducted in vitro susceptibilitytesting for carbapenems, including imipenem, ertapenem, and meropenem, and found that 80% of Klebsiella pneumoniae were resistant to carbapenems.

### Mortality risk factors analyses

The mortality rate of patients with bloodstream infections in ICU was 35.21% (25/71). Univariate analysis of mortality risk factors showed that cardiovascular disease, shock, the use of ventilator and central venous catheter, lower γ-glutamyl transpeptadase, lower prealbumin, elevated mean platelet volume and elevated uric acid levels were associated with the mortality rates (all *p* < 0.05, Table 3). Furmermore, the length of hospital stay, cardiovascular disease, uric acid, mean platelet volume and the use of ventilator were independently associated with mortality during hospitalization.

**Table 3:** Univariate and multivariate analyses of clinical characteristics and risk factors among patients with BSI who died in ICU.

Variable	Univariate analysis			Multivariable logistic regression analysis	
	Survival (n = 46)	Death (n = 25)	*p* value	OR (95% CI)	*p* value
Male	32	18	0.830		
Age	75 (67–84)	74 (64–74)	0.887		
Hospital stay (d)	36 (21–53)	20 (13–53)	0.657	1.025 (1.003–1.048)	0.024*
APACHE II score > 15	27	20	0.070		
Disease factors					
COVID-19	6	6	0.239		
Gastrointestinal	28	15	0.943		
Respiratory	28	16	0.795		
Cardiovascular	15	16	0.011*	6.265 (1.453–27.016)	0.014*
Renal	15	13	0.110		
Neurologic	16	7	0.560		
Metabolic disorders	28	17	0.551		
Immunocompromise	9	3	0.417		
Shock	25	21	0.012*		
γ-glutamyl transpeptadase^a^	71 (33–179)	54 (41–93)	0.022*		
Uric acid^b^	174.1 (121.8–304.9)	314 (191.5–441.4)	0.032*	1.009 (1.003–1.013)	0.004*
Prealbumin (mg/L)	98 (64–98)	77 (37–77)	0.017*		
Brain natriuretic peptide^d^	496 (216–496)	867.8 (263–867.8)	0.277		
Procalcitonin^e^	2.26 (0.72–46.18)	8.59 (4.15–18.69)	0.593		
Hemoglobin^c^	87 (71–87)	79 (65–109)	0.954		
Leukocyte count^f^	8 (6.22–15.44)	10.86 (7.18–20.94)	0.140		
Mean platelet volume (fl)	10.3 (9.5–10.3)	11.3 (10.5–12.4)	0.011*	1.762 (1.09–2.848)	0.021*
CRP (mg/L)	143 (96–143)	126 (76–126)	0.140		
Therapeutic factors					
Surgical intervention	23	10	0.420		
Enteral feeding	9	6	0.662		
Gastrointestinal bleeding	28	16	0.795		
Transfusion	22	17	0.103		
Dialysis therapy	14	15	0.015*		
Tracheotomy	16	9	0.918		
Ventilator	25	22	0.004*	58.857 (3.957–875.57)	0.003*
Central venous catheter	29	24	0.002*		
Antifungal agents	21	9	0.432		
Fluoroquinolone	17	7	0.446		
Peptide antibiotic	18	12	0.470		
Cephalosporin	25	10	0.248		
Carbapenem	31	21	0.131		
Tigecycline	8	5	0.786		
Linezolid	14	7	0.830		

^a^U/L

^b^μmol/L

^c^g/L

^d^pg/ml

^e^ng/mL

^f^10^9^/L

*p < 0.05

## Discussion

Our research investigated the incidence of BSI among ICU patients admitted to our hospital during the latest time period, revealing the infection rate as 10.11%. Compared with other recent domestic studies^10^, the incidence rate of BSI is relatively high and the patients are older which indicates that the population admitted to this hospital has specific characteristics, and the prevention and treatment of BSI in this area might need to be improved. We found that patients with bloodstream infections have longer hospital stays, a higher probability of gastrointestinal bleeding, more frequent use of dialysis treatment and tracheotomy during the treatment process, and the use of tigecycline before positive blood culture. However, the use of antifungal drugs and earlier use of enteral feeding might protect patients from developing BSI. Further multivariate regression analysis revealed that the use of tracheotomy was independently associated with a seven fold increase in the risk of BSI. The risk of tracheotomy ranks first, followed by shock, gastrointestinal bleeding and tigecycline in patients, which implies that early use of tracheostomy in the treatment of patients significantly increases the risk of bloodstream infection. Recent studies have also focused on the use of tigecycline^13^, and some studies have also found a significant correlation between tigecycline use and higher mortality rates in critically ill patients with BSI^13,14^. Therefore, careful attention should be paid to the use of tigecycline in ICU patients. The gastrointestinal tract has long been considered to have an important regulatory role^15^, and BSI patients with gastrointestinal bleeding has higher in-hospital mortality^16,17^. The result of our research also found a significant correlation between gastrointestinal bleeding and the occurrence of BSI, supporting the previous findings.

In our survival analysis of the occurrence of BSI, it was emphasized the benefits of enteral feeding during hospitalization. As the length of hospital stay increases, patients received enteral feeding are less likely to occur BSI, although length of hospital stay is a risk factor for BSI. Several other studies also have suggested a active link between reduced hazard of BSI development and enteral feeding^18,19^. Meanwhile, our survival analysis of the occurrence of BSI also indicates we should be alert to the occurrence of shock.

The distribution of pathogenic microorganisms isolated from blood samples indicates that the highest proportion is Gram negative strains, which is consistent with recent results in some developing^20,21^ and high-income developed countries^3,22,23^. A four-year domestically retrospective study showed that Gram-negative bacteria were most common pathogens among BSI patients, with Escherichia coli being the most frequently isolated pathogen^24^. In contrast, the predominant strains in our ICU were Klebsiella pneumoniae (43.67%) followed by Acinetobacter baumannii (16.9%), which differs from other relevant domestic studies in recent years^24,25^. Therefore, exploring the characteristics of bacterial strains in different regions is urgent for disease prevention and control and our study demonstrated a significant increase in the predominance of Gram-negative bacteria and carbapenem-resistant strains.

The resistance of microorganisms in hospital environments has become one of the most globally public health challenge. In order to prevent and treat infections caused by multidrug resistant pathogens, it is necessary to update the epidemiology of antimicrobial susceptibility to support treatment strategies. In our study, we observed that Klebsiella pneumoniae accounted for the highest proportion had high multidrug resistance, with only high sensitivity to polymyxin b, moderate sensitivity to amikacin and tigecycline^3^. In addition, over 80% of Klebsiella pneumoniae strains exhibitted carbapenem resistant Klebsiella pneumoniae (CRKP), which was one of the major antibiotic resistance types in ICU. This phenomenon may be due to the complex condition of hospitalized patients (delete: and their improper use of antibiotics before admission to the ICU). Therefore, during admission to the ICU, clinical doctors empirically prescribed a wider spectrum of antibiotics and this also highlights the importance of empirical antibiotic selection.

Studies shows that laboratory indicators during the process of disease have a strong correlation with the occurrence of BSI. In this study, we demonstrated that there are usually lower levels of TP, HGB, and PLT, as well as higher levels of PCT and ALP, before the onset of bloodstream infection. Another interesting finding revealed that in addition to the previously reported significant changes in the levels of TP, HGB^26^, and PCT^27^ during the process of BSI, we have paid more attention to changes in platelet count and ALP levels. Platelets are well-known versatile effector cells in hemostasis, inflammation and leukocyte functions. The mechanisms behind thrombocytopenia in BSI were not cleared in current studys. A study from Europe^28^ evaluated the relationship between admission thrombocytopenia and mortality during BSI, suggesting that platelet deficiency can affect the function of leukocyte and enhance endothelial cell activation, leading to impaired vascular integrity and increased risk of death in BSI patients. Our research also supports the statement above, as we observed a sharp decrease of PLT levels in patients with BSI. Although Platelet bacterial interactions have been documented^29,30^, deeper mechanisms need to be studied.

When assessing the potential impact of BSI in mortality, we found that length of hospital stay, cardiovascular disease, the use of ventilator, uric acid and mean platelet volume were independently associated with mortality during hospitalization. A mendelian randomization study^31^ suggested that high uric acid is causally related to adverse cardiovascular outcomes, our patients of ICU with BSI also have similar mortality outcomes. Among BSI patients with concomitant cardiovascular diseases, the risk of death is significantly increased by about 5 times compared to patients without cardiovascular diseases. As for uric acid levels, they were also significantly higher among deceased patients, whic were consistent with other previous studies^32,33^. Therefore, it is important to pay attention to changes in uric acid levels and cardiovascular complications for timely treatment of BSI. Interestingly, we found that PLT decreased significantly when BSI occurred (Table 1) and further analysis (Table 3) of patients with BSI who died revealed a significant increase in MPV. It indicates that in addition to paying attention to platelet count decline, clinical staff are also advised to monitor changes in MPV to enhance their judgment of disease progression. An increase in MPV reflects changes in the size and morphology of platelets, indicating the degree of activation of platelet function and higher inflammatory response^34^. Inflammatory response stimulate varying degrees of platelet activation, leading to changes in platelet parameters^34^. Some studies have proposed using changes in MPV levels as prognostic markers for BSI^35^, but the different pathogenic mechanisms in hematological levels of these biomarkers still need to be further explored. Our study identifies length of hospital stay as a significant risk factor for BSI mortality, with a median length of stay of 20 days among deceased patients. This highlights the importance for clinicians to closely monitor the hospitalization duration of ICU patients and promptly respond during this critical period for optimal care.

Our study has strengths and limitations. Although our hospital is a first-line medical center in suburban areas, the results of a single center may still be susceptible to selection bias. At present, there are few similar studies on BSI patients in other suburban hospitals that can be used as a reference. Next, we will conduct prospective trials to validate the model in multiple centers.

## Conclusions

Our study described the clinical characteristics, prevalent microorganisms and antibiotic susceptibility of BSI parients admitted to ICU of a tertiary hospital in the suburbs of Shanghai. We determined the prevalence rate of BSI in our hospital's ICU was evaluated to be 10.11%. Notably, factors such as tracheotomy, tigecycline usage, gastrointestinal bleeding, and shock during hospitalization are significantly associated with the occurrence of BSI. Additionally, we underscored the advantageous role of enteral feeding. Gram-negative bacteria was found to be the predominant pathogens that cause BSI in ICU, with Klebsiella pneumoniae comprising the majority. In addition, laboratory indicators suggest that except the established markers like PCT and CRP, platelet count may serve as a clinically valuable parameter in promptly identifying high-risk patients and implementing preventive measures. Moreover, focusing on MPV may help evaluate prognosis and requiring further investigation. Consequently, antibiotic therapy management in suburban hospitals were suggested, further strengthening the importance of prevention of the emergence and spread of highly antibiotic-resistant strains in ICU.

## Data Availability

All datasets generated in this study are included in this published article.
